# Pyogenic Granuloma/Peripheral Giant-Cell Granuloma Associated with Implants

**DOI:** 10.1155/2015/839032

**Published:** 2015-12-01

**Authors:** Enric Jané-Salas, Rui Albuquerque, Aura Font-Muñoz, Beatríz González-Navarro, Albert Estrugo Devesa, Jose López-López

**Affiliations:** ^1^School of Dentistry, University of Barcelona, Pabellón de Gobierno, University Campus of Bellvitge, C/Feixa Llarga s/n, L'Hospitalet de Llobregat, 08907 Barcelona, Spain; ^2^School of Dentistry, University of Birmingham, St. Chads Queensway, Birmingham B4 6NN, UK

## Abstract

*Introduction*. Pyogenic granuloma (PG) and peripheral giant-cell granuloma (PGCG) are two of the most common inflammatory lesions associated with implants; however, there is no established pathway for treatment of these conditions. This paper aims to illustrate the successful treatment of PG and PGCG and also report a systematic review of the literature regarding the various treatments proposed.* Methods*. To collect relevant information about previous treatments for PG and PGCG involving implants we carried out electronic searches of publications with the key words “granuloma”, “oral”, and “implants” from the last 15 years on the databases Pubmed, National Library of Medicine's Medline, Scielo, Scopus, and Cochrane Library.* Results*. From the electronic search 16 case reports were found showing excision and curettage as the main successful treatment. As no clinical trials or observational studies were identified the authors agreed to present results from a review perspective.* Conclusion*. This is the largest analysis of PG and PGCG associated with implants published to date. Our review would suggest that PGCG associated with implants appears to have a more aggressive nature; however the level of evidence is very limited. Further cohort studies with representative sample sizes and standard outcome measures are necessary for better understanding of these conditions.

## 1. Introduction

Reactive lesions are characterized as excessive proliferation of connective tissue as a response to chronic irritation [1]. Among these types of lesions, those seen in the oral cavity include pyogenic granuloma (PG), peripheral fibroma, fibroepithelial hyperplasia, peripheral ossifying fibroma, and peripheral giant-cell granuloma (PGCG). PG and PGCG appear to be the ones commonly associated with implants, as in the past few years multiple case reports have been published [1, 2].

PG is defined as an inflammatory hyperplasia that usually appears as a response to irritants, trauma, hormonal changes, or certain medications [3, 4]. Although classically it is called PG, a more correct name would be focal epithelial hyperplasia since the lesion is not strictly a granuloma or an infection [3, 4]. Peripheral giant-cell granuloma (PGCG) is considered a reactive hyperplastic lesion, although its etiology is not entirely known. It is believed that its pathogenesis includes an excessive activation of osteoclasts, which is associated with a proliferation of macrophages, and possibly causes major bone resorption [2, 5].

PG is more frequent in women in their 20s, with a ratio of 3 : 2 [4]. In 75% of cases it occurs in keratinized gingiva, with location in order of frequency of tongue, lips, and then buccal mucosa [3, 4]. It is more common in the maxilla than in the mandible and in anterior as opposed to posterior areas [4]. In contrast, PGCG usually appears in patients who are between their 40s and 60s, and it is slightly more frequent in women and tends to appear more often in the mandible than in the maxilla (Table 1) [4, 5]. The treatment of these lesions generally involves eliminating the irritating factors as well as performing surgical removal [1, 2]. Commonly associated with periodontal disease, where calculus is the irritating factor, surgical removal along with nonsurgical debridement is well described in the literature [6].

Implant rehabilitation has become more common in the last decade and several factors have been studied which could interfere with osteointegration and longevity [7]. Factors such as smoking, diabetes, and periodontal disease have been studied [7–11]. However, with regard to reactive lesions such as PG and PGCG there is no clear pathway for intervention or treatment to manage these lesions and maintain healthy tissue around the implants [7].

The aim of this paper is to demonstrate the successful management of cases of PG and PGCG associated with implants and to review the literature for the various treatment options.

## 2. Materials and Methods

To collect all relevant information about previous published treatments for PG and PGCG involving implants, the authors carried out an electronic search from to January 2000 to June 2015 (Pubmed Central, National Library of Medicine's Medline, Scielo, Scopus, and Cochrane Library) for reactive lesions related to implants (key words: “granuloma”, “oral”, and “implants”). These articles were obtained, and a hand search of their bibliographies identified any pertinent secondary references. This process was repeated until no further new articles could be identified. The inclusion criteria included clinical trials, cohort studies, case-controlled studies, case series, and case reports, published in English, Portuguese, French, and Spanish, which included a clear description of the treatment employed. The search was limited to human studies and all articles which did not fit into the criteria were excluded. The full papers and abstracts identified through the search were independently reviewed by all authors (EJN, RA, AFM, BGM, AED, and JLL) for inclusion in this systematic review. If there was insufficient information provided in the abstract or if there was a disagreement between reviewers, the authors reviewed the full text before reaching consensus through discussion. Data extraction was then completed in duplicate by the same independent reviewers. The following data were collected: study year, gender, age, location of the implant/PG/PGCG, treatment used, relapse, follow-up, and histopathology (Table 2).

The author (JLL) prepared data extraction tables and all authors contributed to summary reports of the selected journal articles and review of the literature.

The case reports protocol was carried out with patient informed consent following guidelines according to the Helsinki Declaration of 1975, as revised in 2000.

## 3. Results

From the 55 articles initially selected, 39 studies were excluded as they were related to teeth or not directly related to implants. All 16 articles selected were case reports; these ranged from reports of a single case to articles describing up to a maximum of 3 cases, such that a total of 21 patients were reported. Of these there were 15 cases of PGCG [2, 5, 9–16, 21] and 6 of PG [4, 14, 17–20] (Table 2). As no experimental or observational studies were identified the authors agreed that it would not be reasonable to critically appraise the quality of the studies and consequently the results are presented from a review perspective.

The majority of patients with PGCG were women (3 : 1) while PG was seen more commonly in males (2 : 1) (Table 2). The average age of the population who suffered PGCG was 49.6 while for PG it was 50.3. The majority of published cases of PGCG associated with implants had suffered bone loss around the implant [2, 5, 11–15], as only 4 of the published cases of PGCG did not experience bone loss [9, 10, 12, 21] (26.7%). On the other hand bone loss did not occur in cases of PG [4, 14, 17–20].

The six cases of PG were treated with excision of the lesion and curettage, though one case involved excision with Er-YAG Laser [18].

The cases of PGCG were treated with a number of different strategies. In all of the cases the lesion was surgically removed, but in addition to this, in nine cases curettage was also performed [2, 4, 8, 9, 11, 14, 21], in two cases the prosthesis was replaced [10, 11], in one case the prostheses were temporarily removed [15], in another case a graft was performed [13], and in four cases the implant was explanted [2, 5, 12] (Table 2).

The 6 cases which suffered relapse all were PGCG. Three of these were treated with excision and curettage only [2, 9, 12], while the other 3 also underwent explantation [2, 8, 12] (Table 2). In fact, from the ones that underwent explantation, one had a PGCG with 8 times of recurrence [12] and the other case which had three times [2] was treated with curettage [2].

### 3.1. Case Report: Pyogenic Granuloma

A 52-year-old male came for consultation reporting two swellings intraorally adjacent to implants that had been placed three years earlier. These lesions had developed over 6 months and were not painful but did bleed on brushing. With regard to his medical history, of interest, he was diagnosed with antiphospholipid syndrome in 2001 and suffered an acute myocardial infarction in August 2011. He also suffered from focal segmental glomerulosclerosis and chronic kidney failure since 2010 but did not require hemodialysis and could be linked to systemic lupus erythematosus. With respect to his dental history, he suffered from advanced chronic generalized periodontal disease and had plaque and calculus deposits both supra- and subgingivally. Due to his advanced periodontal disease the remaining upper and lower teeth had been splinted two years earlier.

The patient's regular medicines included 4 mg acenocoumarol, 100 mg acetylsalicylic acid, pantoprazole 40 mg (proton pump inhibitors), atorvastatin 40 mg (HMG-CoA reductase inhibitors), amlodipine (calcium channel blockers), bisoprolol 5 mg (beta-blocker), and 360 mg mycophenolic acid. No habits of substance abuse were reported.

**Figure 1:**
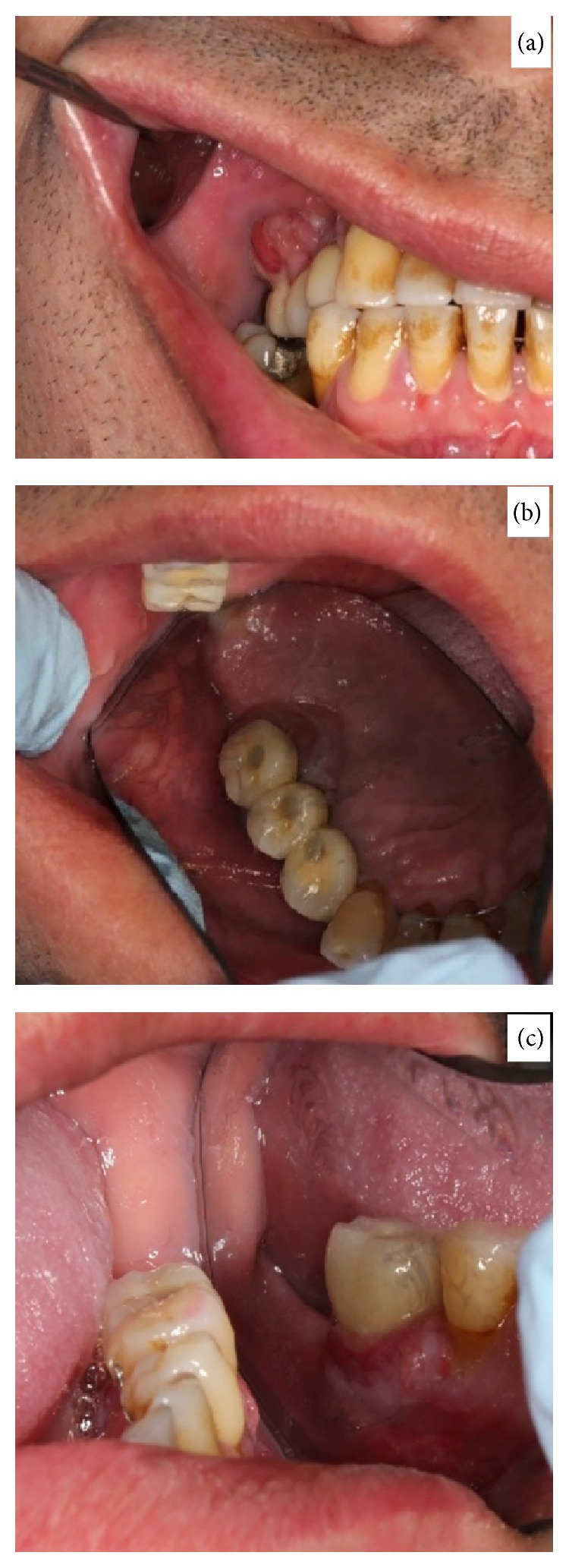
(a) Exophytic granulomatous lesion in 16 and 17 implants. (b) Palatal view of the granulomatous lesion in 16 and 17. (c) Granulomatous lesion in 36.

**Figure 2:**
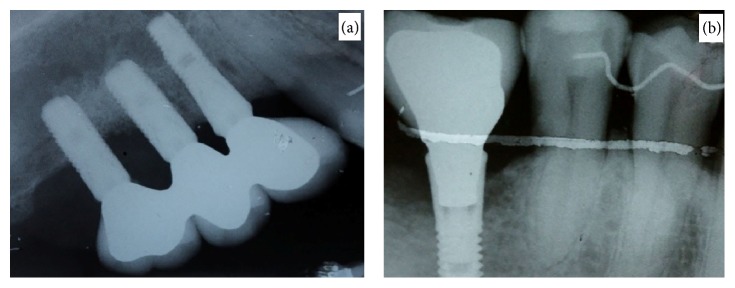
(a) Periapical radiograph that shows bone loss in implants 16 and 17. (b) Periapical radiograph that shows bone involvement in implant 36.

**Figure 3:**
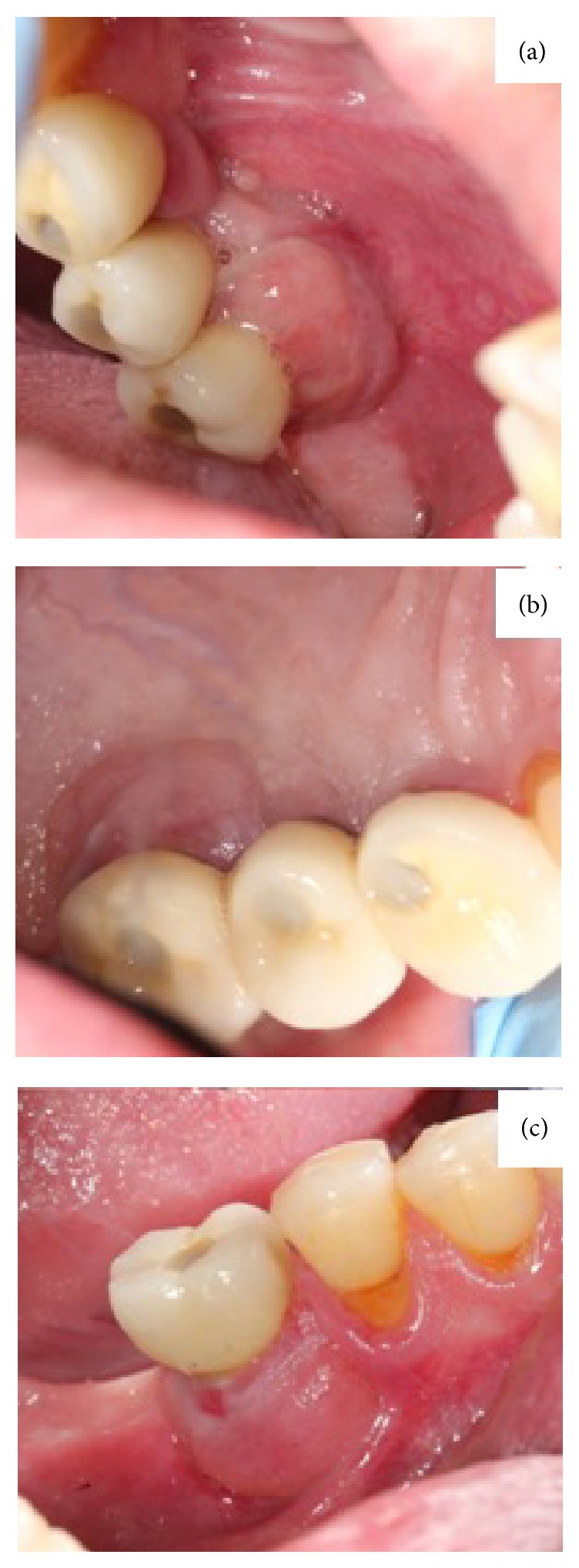
(a) 12-month postexcision follow-up of the lesion in 16 and 17 area. (b) Palatal view of the 12-month postexcision follow-up of the lesion in 16 and 17. (c) 12 month postexcision follow-up of the lesion in 36.

Oral examination revealed two nodular erythematous sessile lumps of 1.5 cm diameter, with elastic consistency and granulomatous appearance. They were associated with the gingiva, located on the buccal and palatal/lingual sides of implants 3.6, 1.6, and 1.7 (Figure 1). Radiographic investigation revealed 5 mm of peri-implant bone loss associated with both lesions. The presumptive diagnosis was that of pyogenic granuloma or peripheral giant-cell granuloma. An excisional biopsy was performed along with curettage and irrigation with chlorhexidine 0.5% of both the surgical site and the exposed implant threads. The microscopic description was that of ulcerated lesions covered by a fibrin and leukocyte membrane and made up of granulation tissue with a mixed inflammatory infiltrate with polymorphonuclears, together with vascular proliferation (Figure 2). Squamous epithelium with parakeratosis, dyskeratosis, acanthosis, and elongation of the epithelial peaks was observed at the far extremes. The epithelial maturation was conserved and no dysplasia-like phenomenon was observed. The results were compatible with a diagnosis of pyogenic granuloma, without any suggestion of malignancy. Postoperatively there were no further issues and there was no evidence of relapse at 12-month follow-up (Figure 3).

### 3.2. Case Report: Peripheral Giant-Cell Granuloma

A 64-year-old male came for consultation regarding a 15-day history of an exophytic mass associated with the buccal marginal gingiva of an implant supported dental prosthesis in the lower right quadrant. This had been placed 8 years earlier. The lesion was asymptomatic, but the patient reported bleeding on brushing.

The patient's medical history revealed complete atrioventricular block, coronary atherothrombosis with acute myocardial infarction in 2006, left ventricular dysfunction, pericarditis, and diabetes mellitus type 2. The patient's regular medicines included carvedilol 6.25 mg, 1/day (beta-blocker), enalapril 5 mg, 1/day (ACE inhibitor), furosemide 40 mg, 1/day (sulphonamide), Tromalyt 150 mg, 1/a day (acetylsalicylic acid), omeprazole 20 mg, 1/day (proton pump inhibitors), and variable dosage of insulin. No habits of substance abuse were reported. With respect to the patient's dental history, he was partially dentate in the mandible as a result of periodontal disease and had multiple implant supported fixed prostheses, with a total of 10 implants placed 8 years earlier.

**Figure 4:**
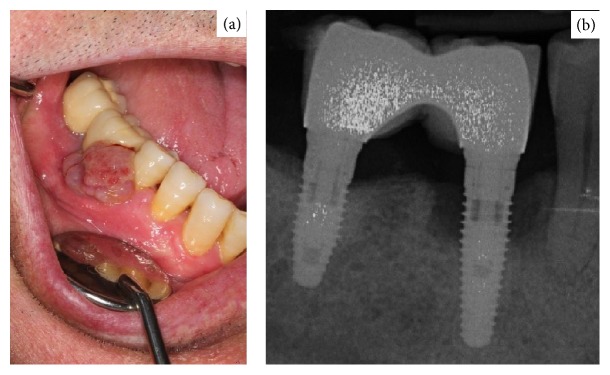
(a) Exophytic granulomatous lesion in 4.6. (b) Periapical radiograph that shows bone involvement in implant 46.

On examination intraorally there was a swelling of 1 cm diameter on the buccal surface of implant 4.6. The lesion was reddish-purple in color and was well defined with an elastic consistency and an irregular texture. It was not mobile and was sessile. Periapical radiograph revealed bone loss of 4 mm affecting both the mesial and distal surfaces of the implant (Figure 4).

**Figure 5:**
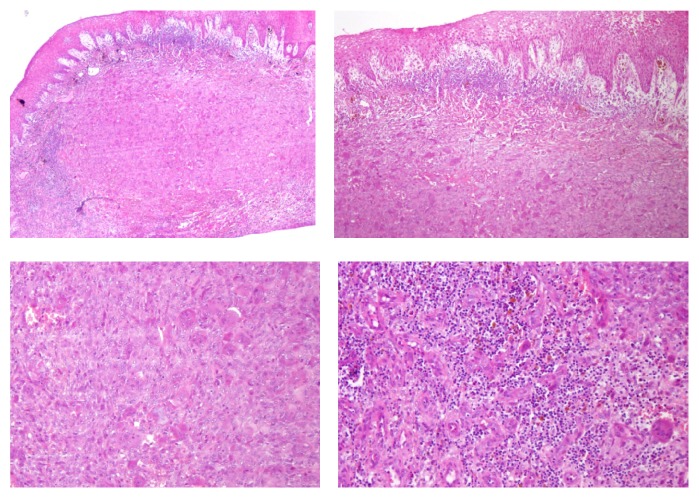
Histopathology of peripheral giant-cell granuloma.

Histopathology confirmed that it was an ulcerated peripheral giant-cell granuloma, without suggestion of malignancy (Figure 5). Histologically it was described as a lesion covered by a parakeratinized stratified squamous epithelium, with areas of atrophy and ulceration in its thickness. A dense proliferation of multinucleated giant cells was dispersed on a stroma of the tissue, which was highly vascularized, with areas of hemorrhage, deposits of hemosiderin, and infiltrate due to accumulation of lymphoplasmacytic inflammatory cells. Laboratory tests showed no abnormalities with regard to calcium/phosphate metabolism or parathyroid gland function.

**Figure 6:**
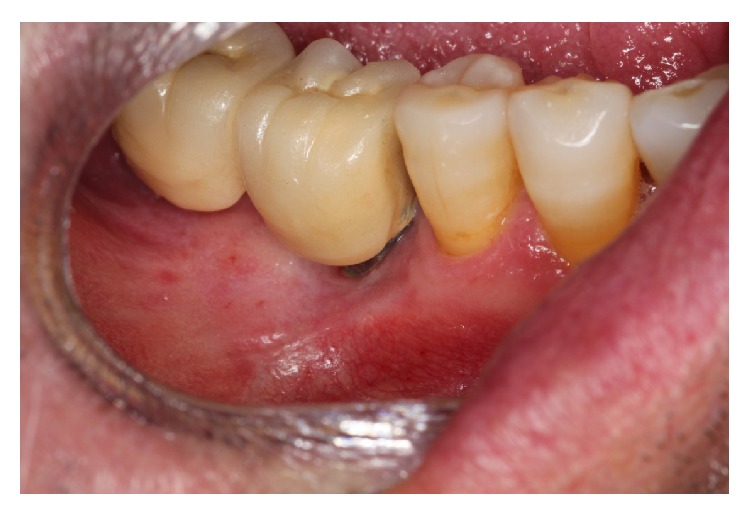
12-month postexcision follow-up of the lesion located in 46.

Treatment involved complete excision of the lesion, curettage of the exposed implant threads, and irrigation with chlorhexidine 0.5%. Postoperative healing was good and at 12-month follow-up there was no evidence of recurrence (Figure 6).

## 4. Discussion

Peri-implantitis with progressive bone loss is reported to be the most frequent complication associated with implants [2]. Its treatment is challenging and to do so correctly we must identify the pathology leading to peri-implantitis [2]. In our view recognition of the soft tissue pathology by biopsy should be the first step for potential successful treatment.

The etiology of these conditions when associated with implants is similar to that which is described when they are associated with teeth, where factors such as trauma, plaque deposits, or chronic infection have a major role [1]. In cases associated with implants, incorrect or inadequate prosthesis (implant cap or healing cap, poorly adjusted suprastructures, etc.) is also considered to be a possible causative factor [2, 9]. With regard to inadequate prosthesis, Bischof et al. [10], Ozden et al. [11], and Peñarrocha-Diago et al. [15] replaced the prosthesis or temporarily removed the prosthesis, allowing for better plaque control, and they reported no recurrence. Another suggested potential cause, although controversial, is that an inflammatory response to titanium may lead to development of granulomas [10, 12–15, 22].

The clinical presentation and age group of the PG and PGCG cases described appeared to be similar when associated with teeth [23–27]. Our data collection showed PGCG is more frequent in women and in the posterior mandible [2]. One could propose this may be due to greater plaque accumulation posteriorly due to difficult access for thorough oral hygiene. When associated with teeth, PG is also commonly seen in females [1, 2]. This is in contrast with our findings which suggest that when associated with implants it is more prevalent in men. Nonetheless this may be an inaccurate conclusion and due to the small number of cases reported.

In females, pregnancy can have a role in this condition. It has been suggested that the increase of estrogen and progesterone can influence gingival physiology, enhancing the tissue response to the local microbiota, with a predominance of more pathogenic microorganisms [20, 27, 28].

Data from the selected articles suggests that bone loss from the implant site is more commonly associated with PGCG [2, 5, 11–15] than PG. Hernández et al. [2], Cloutier et al. [5], and Bischof et al. [10] support the hypothesis that bone loss occurs first, exposing the implant collar, which then contributes to irritating factors that lead to PGCG. This is also more prevalent when lesions present posteriorly. Again this could be related to difficult access for thorough oral hygiene though this may also be due to the greater occlusal load experienced posteriorly, as opposed to that experienced by the anterior teeth [2, 5, 10].

The prevalence of recurrence in cases of PG and PGCG is estimated to be 2.9–8.2% and 5–11%, respectively, but in cases associated with implants these figures increase [2, 9, 16, 17, 21]. Recurrence has been reported in 6 of the 15 published cases of PGCG associated with implants (Table 2). Lester et al. [23], in a review of 279 cases, noted a total of 10 recurrences on 5 implant cases; 1 implant case had 2 recurrences, and another case with multiple implants had 8 recurrences. It was noted that while 2 of the 5 implant cases (40%) had multiple recurrences, 6 of 237 non-implant-related cases (2.5%) presented with multiple recurrences too. Several possible explanations are considered, such as incomplete excision, but in addition these lesions may have been caused by combination of irritating factors, which therefore makes it challenging to eradicate all possible causes [2, 5, 8].

Several treatments have been described when treating PG/PGCG when associated with implants, with excision and curettage being the most common. This is consistent with normal convention when these lesions are associated with teeth (being a relatively simple technique and with satisfactory results [1, 2, 9, 10, 12, 25, 26, 29]).

Other options have been described such as the use of Er-YAG Laser [18] or explantation of the implant and the provision of a new prosthesis [10, 11] or indeed temporary removal [15]. It is suggested that use of the Er-YAG Laser for granuloma excision offers advantages in comparison to conventional surgery techniques, especially by reducing the risk of bleeding, pain, and postoperative edema and also eliminating the need for sutures at the end of the procedure [18].

Given the small number of cases published, it is difficult to evaluate if explantation of the implant affects the number of recurrences or the amount of bone loss. Recurrences of PG and PGCG have been described when associated with bad plaque control but also due to hormonal imbalance during pregnancy, especially in PG cases [30]. The authors believe that more aggressive treatments such as explanation can be used as a secondary technique, only after excision and curettage have failed as explantation could be beneficial in improving plaque control and consequently reduce the number of relapses and amount of bone loss. However the data available about its use in implants is still limited. The use of antimicrobials such as chlorhexidine, as described in our cases, is reported to offer improved plaque control in the treatment of peri-implantitis [29].

## 5. Conclusion

In conclusion, we believe the primary approach to manage these two soft tissue conditions should be excisional biopsy and subsequent histopathology. The authors believe oral hygiene instruction should be one of the first steps in management as good plaque control could help reduce number of recurrences. This review of the literature highlights that histopathological diagnosis is important, as if PGCG is diagnosed histologically, then the clinician will be aware of a higher risk of bone loss and higher rate of recurrence. This would highlight the need of close monitoring and regular review. However, we recognize the limitations of this analysis due to the limited number of case reports published and consequently these conclusions should be interpreted with caution. Further cohort studies with representative sample sizes, control group, and standard outcome measures are necessary.

## Figures and Tables

**Table 1:** Summarizing the differential diagnosis between pyogenic granuloma and peripheral giant-cell granuloma.

	Pyogenic granuloma	Peripheral giant-cell granuloma
Age	20s	40s–60s
Sex	Women	Women
Localization	Anterior maxilla	Posterior mandible
Symptomology	Asymptomatic	Asymptomatic
Color	Reddish	Reddish-purple
Sessile/with a pedicle	Both	Both
Bone involvement	No	Possible

**Table 2:** Most highlighted characteristics from published cases of PG and PGCG associated with implants.

Author (year)	Sex	Age	Localization	Bone loss	Final treatment	Relapse	Follow-up (months)	PG/PGCG
Hanselaer et al. (2010) [9]	F	34	Posterior maxilla	No	Excision + curettage	1	8	PGCG
	M	31	Posterior mandible	Yes	Excision + curettage	1	—	PGCG
Hirshberg et al. (2003) [12]	F	69	Anterior maxilla	No	Excision + explantation	1	—	PGCG
	M	44	Posterior mandible	Yes	Excision + explantation	8	—	PGCG
Bischof et al. (2004) [10]	F	56	Posterior mandible	No	Excision + new prosthesis + control of plaque	No	36	PGCG
Scarano et al. (2008) [13]	F	48	Posterior maxilla	Yes	Excision + soft tissue graft	No	—	PGCG
Cloutier et al. (2007) [5]	M	21	Posterior mandible	Yes	Excision + explantation	No	12	PGCG
	F	62	Posterior mandible	Yes	Excision + curettage	No	2	PGCG
Hernández et al. (2009) [2]	F	45	Posterior mandible	Yes	Excision + curettage	5	108	PGCG
	F	36	Posterior maxilla	Yes	Excision + explantation	3	12	PGCG
Ozden et al. (2009) [11]	F	60	Posterior mandible	Yes	Excision + new prosthesis	No	12	PGCG
Olmedo et al. (2010) [14]	F	64	Anterior maxilla	Yes	Excision + curettage	No	24	PGCG
	F	75	Posterior mandible	No	Excision + curettage	No	48	PG
Dojcinovic et al. (2010) [4]	M	32	Posterior maxilla	No	Excision + curettage	No	18	PG
Peñarrocha-Diago et al. (2012) [15]	F	54	Posterior mandible	Yes	Excision + curettage + temporary removal of the prosthesis	No	12	PGCG
Galindo-Moreno et al. (2013) [16]	M	74	Anterior maxilla	No	Excision	No	6	PGCG
Etöz et al. (2013) [17]	M	55	Posterior mandible	No	Excision + curettage	No	8	PG
Kaya et al. (2013) [18]	M	39	Posterior mandible	No	Excision with an Er-YAG Laser	No	6	PG
Kang et al. (2014) [19]	M	68	Posterior maxilla	No	Excision + curettage	No	12	PG
Trento et al. (2014) [20]	F	33	Posterior mandible	No	Excision + curettage	No	6	PG
Brown et al. (2015) [21]	F	46	Posterior mandible	No	Excision + curettage	No	6	PGCG

M: male; F: female; PG: pyogenic granuloma; PGCG: peripheral giant-cell granuloma.
